# Depression, social support and associated factors among women living in rural China: a cross-sectional study

**DOI:** 10.1186/s12905-015-0180-7

**Published:** 2015-03-21

**Authors:** Fengsu Hou, Catherine Cerulli, Marsha N Wittink, Eric D Caine, Peiyuan Qiu

**Affiliations:** Department of Epidemiology and Statistics, West China School of Public Health, Sichuan University, No.17, 3 section South Renmin Road, Chengdu, Sichuan 610041 China; Injury Control Research Center for Suicide Prevention, University of Rochester Medical Center, 601 Elmwood Ave, Box PSYCH, Rochester, NY 14642 USA; Department of Psychiatry, University of Rochester Medical Center, 601 Elmwood Ave, Box PSYCH, Rochester, NY 14642 USA

**Keywords:** Depressive mood, Social support, Females in rural China, Mental health

## Abstract

**Background:**

Few studies have focused on depression and social support in Eastern populations, especially women in rural China. Our research investigated depression among women in rural China, and studied the relationships between social support and depression.

**Methods:**

We recruited women ages 16 years and older from north Sichuan. Participants completed socio-demographic measures, the Center for Epidemiologic Studies Depression Scale, and the Duke Social Support Index. The analysis method included descriptive statistics and logistic regression.

**Results:**

The final sample included 1,898 participants with a mean age of 48.6 years, and the prevalence of significant depressive symptoms was 12.4%. Results suggest being unemployed, having poorer perceived health/economic status, and lower social support were positively associated with depression. Younger age and greater social support were negatively associated with depression.

**Conclusions:**

This study provides insights on the psychological health of women in rural China and potential directions for future research. These issues are especially pertinent during this time of rapid economic transformation and outmigration in rural China.

## Background

We report findings from a study of the relationship between distress, depression, and social support among women residing in rural China. In the context of China’s extraordinary economic transformation, rural women continue to serve as primary care givers for their families and are often “left behind,” as their spouses seek employment in urban centers and industrial zones. We recently conducted a structured epidemiological survey that found higher rates of distress and depression than reported previously, and it was our impression that these results reflected, in part, our selection of a study region where a fairly high percentage of participants were “left behind”; that is, relatively older, less educated women whose spouses were away from the villages we sampled. This paper grows from that work.

Researchers from a study in Western China suggested that women with husbands who were migrant workers were more likely to develop psychological problems, including depression and anxiety [1]. In the 1990s, unipolar major depression was the second largest contributor to the total disease burden in China, with prevalence rate of 6.2% [2], even though the reported prevalence of depression in China was lower than in other countries [3]. Although China has not conducted national prevalence studies, regional studies have indicated that women report greater mental health burdens than men. Hu and Lu’s research reported the prevalence of depression was 1.2% in Jiangxi Province in 2003, and the odds ratio (OR) between women and men was 1.91 [4]. Kunming reported the prevalence rates of lifetime depression in women and men were 2.4% and 1.5% respectively in the capital of Yunnan Province in the southwest of China [5], using the Composite International Diagnostic Interview (CIDI). In 2009, Phillips and colleagues reported that mood disorders were more common in women than men, including major depressive disorder, dysthymic disorder, and mood disorder not otherwise specified (MD-NOS), and the prevalence of mood disorders in women and in men were 7.3% and 5.0% respectively [6]; simultaneously, they reported that depression was a risk factor for suicide [7]. In 2002, the National Health and Family Planning Commission of the People’s Republic of China (NHFPC), formerly the Chinese Ministry of Health, reported that suicide was the fifth leading cause of death, resulting in 287,000 deaths per year, and the risk factors of depression and subsequent suicide are more prevalent for rural Chinese women than women living in urban areas [8,9]; in 2012, the suicide rate among rural women was 8.58 per hundred thousand people [10,11]. Our research found that 7.5% of women living in rural Sichuan reported significant distress, as detected by the Center for Epidemiologic Studies Depression Scale (CES-D), and of these, 84.6% had a diagnosed mood disorder or other DSM diagnosis (Qiu P, Caine ED, Hou F, Cerulli C, Wittink MN, Li J, Zhang Y: The prevalence of distress and depression among women in rural Sichuan Province, submitted).

Greater social support has been shown to protect people from physical and mental health problems, and can prevent depression in the face of life adversities [12-14]. Social support can be defined as a combination of people’s social networks, and the quality of their formal or informal interactions with other people or groups; further, social support is central to obtaining and maintaining one’s self-value, material, information, and emotional support [15]. Shumaker and Brownell suggest social support has to be perceived by the provider or the recipient, and the value of the relationship is related to the satisfaction of being happy, healthy and prosperous [16]. Social support plays a critical role in providing the context for promoting individual psychological health [17-19], and epidemiological studies suggest that persons with low levels of social support have higher mortality rates [20]. As noted by Berkman and Syme, social support is potentially linked to health outcomes through several pathways, including psychological processes [21].

In this study we used the Buffer Theory as a framework for studying social support. The Buffer Theory, formed in the 1970s, has been the foundation of many studies [22]. Cassel suggested that people could benefit from social support to reduce or buffer the stresses of daily life, or the potential impact of adverse life events, such as depression [22]. Others have supported this contention [23,24]. In addition, we focused on the three subtypes of social support that Cohen conceptualized [25]: 1) social interaction, support received from social networks; 2) perceived social support, conceptualized and/or recognized availability of social support; 3) instrumental social support, tangible aid such as financial and material resources. While research about social support has focused upon Western populations, there have been few studies among Asian samples—especially residents of rural China.

Hence, we hypothesized: 1) social support would be associated with depression in rural China; 2) the higher social support participants received or perceived, the less likely they would be depressed.

## Methods

### Sample and sampling

We conducted a cross-sectional study focused on the towns and areas surrounding Guangyuan City, in northern Sichuan Province near the border with Shaan Xi Province. Of note, a “city” in China most often is a region that can include multiple counties, towns, and villages, including broadly ranged rural areas. Guangyuan City is not a major, highly modern urban-suburban area, such as Sichuan’s capital, Chengdu City, nor is it part of the coastal industrial expansion that powered China’s economic revolution. The region is economically under-developed; in 2010, its per capita GDP of RMB 12,313 Yuan (approximately 2,028 US dollars), was the second lowest in Sichuan Province [26].

As described in greater detail elsewhere (Qiu P, Caine ED, Hou F, Cerulli C, Wittink MN, Li J, Zhang Y: The prevalence of distress and depression among women in rural Sichuan Province, submitted), we used multistage sampling to select towns and villages for the study, assuring participant diversity in terms of socio-economic factors. At first, we selected three towns by random sampling; then, we randomly sampled three to five villages for each town, and eventually we selected 13 villages.

### Procedure

For this study we included all women, ages 16 years and older, who had been living in the selected villages and towns for at least 2 years. Sixteen years is the designated age for adult consent in China. We used the Chinese household registration system (the *hukou*) to identify eligible women. Participants were compensated for their time with toiletry items (such as toothpaste and soap) worth 5 Yuan (about 0.8 USD). Based on registration lists provided by local hospitals, we excluded women if 1) they were disable to communicate with interviewers; 2) they had a previous diagnosis of any severe mental or cognitive problems, which would impede their ability to comprehend the questions. The full excluded conditions were deaf, mute, schizophrenia, paranoia, autism, dementia and mental retardation. Considering rural Chinese women usually have low education and could be illiterate, we provided verbal informed consent without an information sheet to the participants. Participants provided answers only if they orally consented to the survey during a face-to-face interview in their houses.

The protocol including verbal the informed consent was reviewed and approved by the Ethics Committee of Sichuan University. The University of Rochester Research Subjects Review Broad reviewed and approved analyses of de-identified data.

### Instruments and measurements

#### Demographic information

We collected demographic information, such as: age, nationality, marital status, education, occupational status, perceived family financial condition and personal health status.

#### CES-D scale

The Center for Epidemiologic Studies Depression Scale (CES-D) is a self-report inventory for use in studies of the epidemiology of depressive symptomatology and expressions of distress [27]. The English version and Chinese version of CES-D have been used among the Chinese for years [28-30]; its validity and reliability have been established [29]. This study used a cut-off point of 15/16, judging a score between 16 and 20 to be indicative of distress or depression symptoms, and a score of ≥21 as consistent with a clinical diagnosis of depression or another condition [27,30].

#### Duke social support index

The Duke Social Support Index (DSSI; [31]) was originally a 35-item scale used to explore the interactive effects of life events and social support on major depressive episodes and depressive symptoms. We used the 23-item version of the DSSI in this study, which captures the essential components of social support related to mental health outcomes [32]. The Chinese version has already been applied in research [33,34], and studies in Chinese rural samples reported its internal consistency was over 0.79 [35,36]. Consistent with our interests, the 23-item DSSI investigates social support from three dimensions: social interaction, perceived social support and instrumental social support. Every answer has been assigned a score, and the total reflects the sum of the items (possible total scores ranged from 11 to 45).

### Procedure

We conducted the field survey in July 2012. Local government and Guangyuan Mental Health Center staff helped recruit participants. They coordinated with village leaders and village doctors, and we held public information sessions about our study in villages before the survey began. Once the survey began, village leaders, doctors and seniors led interviewers door-to-door to conduct the interviews. As some villages in Guanyuan have low population density, local residents helped interviewers by transporting them door-to-door with motorcycles. When an eligible participant was not at home, or not available for the interview, the interviewer would return twice to try and recruit the participant.

Interviewers conducted the surveys on their own personal computers during the face-to-face interviews. Data were deleted from interviewers’ personal computers after being copied to the research leader’s flash drive.

### Quality control

Details of training and quality control measures are reported elsewhere (Qiu P, Caine ED, Hou F, Cerulli C, Wittink MN, Li J, Zhang Y: The prevalence of distress and depression among women in rural Sichuan Province, submitted). In brief, interviewers were recruited from among medical students from West China School of Public Health of Sichuan University. Faculty from West China University, the University of Rochester Medical Center, and Zhejiang University conducted training sessions related to study methods.

We deployed three research teams; each was comprised of eight student-interviewers and led by experienced senior researchers. Questionnaires were routinely checked for missing items.

### Analysis

We applied bivariate logistic regression to analyze demographic characteristics, social support, and the results of the CES-D test, and chose multi-stage logistic regressions to explore the fit model among depression, social support and other factors. We also used one-way ANOVA and Chi-square tests to compare CES-D and DSSI scores between women with and without migrant husbands.

We categorized participants into four groups based on age: ≤ 24 years old, 25–44 years old, 45–64 years old, and ≥ 65 years old. We divided participants into five education groups: lack of formal schooling (no school), primary level of education (≤6 years), junior high school level of education (7–9 years), high school level of education and above (≥10 years). Occupational status was categorized into four groups: agriculture work, non-agriculture work, homemakers/students, and unemployed. In addition, we categorized subjects into four groups based on their perceived yearly household income: affluent, basic enough, some difficulty, very difficult. Perceived health status was categorized into four groups: very good, good, adequate, and poor. We dichotomized marital status into two groups: married and not married. Based on the distribution of the scores of DSSI, the subjects were divided into three groups: high, medium, and low social support, and the cut points were 25% and 75%. In our study, statistical significance was established at *p* < 0.05.

## Results

We recruited 1,919 consenting participants from whom we obtained 1,898 completed surveys for the current analyses.

### Demographic characteristics of subjects

The ages of participants ranged from 16 to 90 years, with a mean of 48.6 ± 15.5 years; 1556 (82.0%) were married. The majority of the women in our sample were not well educated—724 (38.2%) of them had never been educated, 706 (37.2%) of them had received less than six years education, and 324 of them (17.1%) had received seven to nine years education; only 144 of them (7.59%) had been educated for 10 or more years. Occupational status included: agricultural workers – 1117 (58.9%); non-agricultural workers – 185 (9.75%); homemakers/students – 379 (20.0%); and unemployed – 179 (9.43%). Results regarding household income revealed: 981 – (51.7%) women stated their household income was sufficient; 596 (31.4%) stated that the family had some difficulty covering their expenditures; and 73 (3.85%) stated it was very difficult for them to cover their expenditures. Results regarding perceived health status revealed: 287 (15.1%) women stated their health was very good; 389 (20.5%) and 696 (36.7%) described theirs as good or adequate, respectively; and 525 (27.7%) defined their health status as poor.

### The relationship between social support and depression/distress

#### Bivariate logistic regression

The prevalence of depression/distress in our study was 236/1898 (12.4%) as defined by a score of 16 or more. We calculated odds ratios using a bivariate logistic regression model to explore the strength of the relationship.

Results revealed the odds ratios (ORs) for the factors: aged 45 to 64 years old 2.13 (95%CI: 1.12-4.05, compared to aged 16–24 years old), unemployed 1.62 (95%CI: 1.08-2.43, compared to agriculture work), adequate health status 3.84 (95%CI: 1.90-7.78, compared to very good health status), poor health status 10.2 (95%CI: 5.09-20.3, compared to very good health status), some difficulty in household income 3.19 (95%CI: 1.79-5.71, compared to affluent household income), very difficult in household income 13.0 (95%CI: 6.40-26.5, compared to affluent household income), medium social support 1.72 (95%CI: 1.15-2.57, compared to high social support), low social support 4.26 (95%CI: 2.83-6.41, compared to high social support), and received education 10 years and above 0.36 (95%CI: 0.17-0.75, compared to no school). See Table 1.

**Table 1:** **Bivariate logistic regression of depression**

**Participants’ characteristics**	**Total number (1898)**	**Depression**		
		**No (1662)**	**Yes (236)**	**OR (95%CI)**
Age				
16-24*	143 (7.53%)	132	11	----
25-44	605 (31.9%)	544	61	1.35 (0.69-2.63)
45-64	843 (44.4%)	716	127	2.13 (1.12-4.05)**
65-	307 (16.2%)	270	37	1.64 (0.81-3.33)
Marital status				
Single*	342 (18.0%)	292	50	----
Married	1556 (82.0%)	1370	186	0.79 (0.57-1.11)
Education				
No school*	724 (38.2%)	622	102	----
Less than 6 years	706 (37.2%)	615	91	0.90 (0.67-1.22)
7 to 9 years	324 (17.1%)	289	35	0.74 (0.49-1.11)
10 years and above	144 (7.59%)	136	8	0.36 (0.17-0.75)**
Occupation***				
Agriculture work*	1117 (58.9%)	971	146	----
Non-agriculture work	185 (9.75%)	169	16	0.63 (0.37-1.08)
Homemakers/students	379 (20.0%)	342	37	0.72 (0.49-1.05)
Unemployed	179 (9.43%)	144	35	1.62 (1.08-2.43)**
Perceived health status***				
Very good*	287 (15.1%)	278	9	----
Good	389 (20.5%)	369	20	1.67 (0.75-3.73)
Adequate	696 (36.7%)	619	77	3.84 (1.90-7.78)**
Poor	525 (27.7%)	395	130	10.2 (5.09-20.3)**
Perceived household income				
Affluent*	223 (11.8%)	209	14	----
Basic enough	981 (51.7%)	901	80	1.33 (0.74-2.39)
Some difficulty	596 (31.4%)	491	105	3.19 (1.79-5.71)**
Very difficult	73 (3.85%)	39	34	13.0 (6.40-26.5)**
Social support***				
High*	531 (28.0%)	496	35	----
Medium	923 (48.6%)	823	100	1.72 (1.15-2.57)**
Low	433 (22.8%)	333	100	4.26 (2.83-6.41)**

*Reference group; **p <0.05; ***Participants who were not responsive were coded as missing data.

#### Social support in depressed and non-depressed groups

We assessed social support by examining social interaction, perceived social support, and instrumental social support, and compared social support levels between depressed and non-depressed groups based on CES-D results. Table 2 shows that subjects in the non-depressed group had statistically higher DSSI scores in all three dimensions.

**Table 2:** **Subgroups of social support and depression**

**Social support**	**Mean scores in depression groups**			
	**Depressed**	**Not depressed**	**F**	***p***
Social interaction	7.31 ± 1.70	7.80 ± 1.74	16.2	<0.05
Perceived social support	17.6 ± 3.33	19.0 ± 2.70	54.6	<0.05
Instrumental social support	9.61 ± 3.14	10.8 ± 2.26	47.7	<0.05

Note. ANOVA was applied to test the statistic significant differences of social support between the groups.

#### Multi-stage logistic regression

To explore our hypothesis and examine the contribution of social support in decreasing depressive symptoms, we controlled for demographic variables and added social support variables stepwise into the model, shown in Table 3.

**Table 3:** **Multi logistic regression model between social support and depression**

**Participants’ characteristics**	**Block 1**		**Block 2**		**Block 3**	
	**OR (95%CI)**	***P***	**OR (95%CI)**	***P***	**OR (95%CI)**	***P***
Perceived social support	0.90 (0.86-0.94)	0.00	0.90 (0.86-0.94)	0.00	0.94 (0.89-0.99)	0.01
Social interaction	----	----	0.97 (0.88-1.06)	0.48	0.98 (0.89-1.08)	0.67
Instrumental social support	----	----	----	----	0.94 (0.87-0.97)	0.002
**Controlling factors**						
Perceived household income	1.63 (1.34-1.98)	0.00	1.62 (1.34-1.97)	0.00	1.64 (1.34-2.00)	0.00
Perceived health status	1.87 (1.56-2.24)	0.00	1.87 (1.56-2.24)	0.00	1.87 (1.56-2.24)	0.00
Age	0.90 (0.71-1.13)	0.36	0.89 (0.70-1.12)	0.32	0.88 (0.70-1.11)	0.28
Education	1.09 (0.87-1.36)	0.46	1.10 (0.88-1.38)	0.40	1.10 (0.88-1.38)	0.42
Marital status						
Single*	----	----	----	----	----	----
Married	0.82 (0.56-1.21)	0.32	0.82 (0.56-1.21)	0.32	0.81 (0.55-1.20)	0.30
Occupation						
Agriculture work*	----	----	----	----	----	----
Non-agriculture work	0.81 (0.44-1.50)	0.51	0.82 (0.45-1.51)	0.52	0.84 (0.46-1.54)	0.57
Homemakers/students	0.77 (0.51-1.16)	0.21	0.77 (0.51-1.16)	0.21	0.76 (0.50-1.16)	0.20
Unemployed	1.32 (0.84-2.07)	0.24	1.31 (0.83-2.06)	0.24	1.34 (0.85-2.11)	0.21
Constant	0.023	0.00	0.028	0.00	0.032	0.00
**Omnibus tests of model coefficients**						
Model Chi-square	169.2	0.00	169.7	0.00	178.6	0.00
**Hosmer-Lemeshow Test** (*χ* ^2^)	11.6	0.17	11.9	0.16	13.8	0.09

Note. The analysis included 1826 cases. *Reference group.

When the three dimensions of social support were included in the regression model, the Hosmer-Lemeshow Test indicated that Model 1 offered the best fit with the smallest chi-square value 11.57. The results revealed that perceived social support remained robust in all the models. Model 3 suggested that instrumental social support was negatively associated with depression (OR = 0.94, 95%CI = 0.87-0.97). Perceived household income and perceived health status were extremely important factors related to depression, even after being controlled in Model 1. The worse the participants perceived their life circumstances, the more likely they were to suffer from depression and the ORs were up to 1.63 and 1.87 respectively.

### The influence of being left-behind

Among 1,556 married women, 1,537 of them answered the question about their husbands’ migration status. In total, there were 240 women with migrant husbands. The results showed that there were not significantly different mean scores of DSSI between women with and without migrant husbands (37.4 ± 4.86 vs. 37.3 ± 5.21, *p* > 0.05). The scores of three social support subgroups did not show significant differences either.

The mean scores of the CES-D for women with and without migrant husbands were 6.49 ± 8.44 and 6.90 ± 8.24 (*p* < 0.05). Meanwhile, the distribution of screening positive for depression did not show any differences between the two groups. The table was not shown in this paper.

## Discussion

This study focused on women from rural Sichuan where social and economic conditions are much less developed compared with many provinces in Eastern China. Participants included grandmothers who take care of their retired husbands and grandchildren; women who care for parents, in-laws, and children; and unmarried, divorced, and separated women. This study assessed the relationship between social support, as well as other factors, and depression/distress. Even though urbanization has begun, the economic conditions in regions similar to our study site remain far worse when compared to settings where economic changes began two to three decades ago.

In our study, the prevalence of depression/expressed distress was 12.4%, higher than previous studies [3-5] that used either the Structured Clinical Interview for DSM-IV (the SCID) or the CIDI to establish a psychiatric diagnosis. The CES-D is not a clinical diagnostic tool, rather it is a self-report inventory that uses a continuous scale to define levels of depressive symptoms. As we discussed in our initial work, it may be more sensitive to reports of significant distress that do not conform to specific diagnostic symptom categories.

Consistent with previous findings that underscore the important role of social support in the context of depression, we found that greater support was apparently protective while diminished levels were associated with an increased expression of symptoms [13,14,37,38]. The prevalence of elevated symptoms of distress/depression was four times higher among women with low social support. Additionally, the domain of perceived social support played a more important role than the other two subtypes. Traditionally, China has been a very social society, especially in rural regions where assisting others was common during planting and harvest times, and when dealing with environmental crises [39-41]. The economic transformation of the past three decades has challenged the family-focused basis for social cohesion, but most villagers responding to our inquiries expressed that they trusted and were willing to assist their neighbors. This willingness can be considered as instrumental social support to others, and also can turn into perceived social support when the villagers know they can get support from others.

This deeply rooted Chinese tradition provided insight on the future health care model. Residents in rural regions were encouraged to enroll in the New Cooperative Medical Scheme (NCMS), which focuses on protecting them from becoming impoverished by critical health expenses [42]. This community-based health care system provided health insurance for patients with severe persisting psychosis [43], and now the system has begun to pay attention to more prevalent conditions like distress, depression and anxiety. Considering the virtue of helping others in Chinese culture as a component of a health care model, along with improving social support within a rural community, perhaps we can not only reduce the risk of depressive symptoms, but also refine the NCMS by motivating rural residents to participate in the transition.

Similar to previous studies, which indicated lower income was associated with elevated risk of depression [44-46], we found aggravated perceived economic status were associated with an elevated CES-D score among our sample of rural Chinese women. A systematic review reported self-rated health status was associated with the risk of depression [47], which was consistent with our findings that perceived health status was strongly associated with depressed symptoms. As to perceived health status, a study in Shanghai reported poorer perceived health status was associated with higher mortality risk [48].

Thus, we hypothesized and continue to maintain that the perception of oneself, regardless of its content, was critical to health.

While some studies suggest that lower education is associated with depression [49-52], we found no effect in our sample, which was so heavily weighted for low education (92.4% of participants had less than 10 years) that we did not have sufficient variance in the sample to demonstrate any possible influence. We know that more educated women in our target villages tended to become migrant workers, thus skewing those whom we studied.

Women living in rural China often bear the double burden of managing a family and performing the bulk of the family’s agricultural work. This may serve as a primary income source in some families, not simply a supplement to their husband’s contribution. Indeed, researchers have characterized women’s participation in farming as the feminization of agriculture [53,54]. In such situations, the availability of practical day-to-day support from family and neighbors during times of stress may mitigate the impact of stressful or adverse life events, provide alternative solutions to problems and prevent the development of symptoms or dysfunction; it may also aid in dealing with those symptoms should they develop. However, the design of our study limited our ability to make definitive interpretations. We encourage future research to ascertain how such social support may operate – whether it diminishes the frequency of adverse effects or reduces daily stress levels, or aids in response to challenges by sharing the burden of problems (“dealing with *ou*r problem *together*”), or serves to bolster individuals as they address their concerns (“help you deal with *your* problem”).

Marriage often is viewed as a protection against mental distress or disorders [55-58]. We found married women were more likely than their unmarried peers to rate higher levels of social support. However, we caution about interpreting this finding, given that 82.0% of our sample was married, limiting the potential variance of our data. Similarly, we did not find that “left-behind” status influenced psychological well-being, despite our assumption when we began the study. Again, we realize that there were a relatively small number of women who had migrant husbands, limiting our ability to detect smaller effects. However, it was clear that what we had anticipated as a powerful influence was not evident.

Women aged 45 to 64 years were more likely to show depressive symptoms than the youngest group in bivariate regression. Collectively, these rural middle-aged women were characterized by lower education, low income, performing agriculture work, and more dependent on their own and/or their spouses. These results suggest that future programs to reduce depression and prevent suicide should especially focus on middle-aged women in rural China.

Currently, the NHFPC is developing new initiatives to begin the process of addressing more common mental disorders, such as depression; this effort is related, in part, to China’s new mental health law and further development of its rural health insurance systems, and to the realization that much of the burden of disease relates to non-psychotic conditions [59]. These efforts in rural regions necessarily will focus on educating and supporting village and town doctors, most of whom have scant knowledge of mental health conditions. Based on our findings, we recognize a need to address common factors that appear to contribute to or be associated with the genesis of depression and distress: being middle aged, unemployed, having financial difficulties, having poor perception of self-health status, and feeling more socially isolated or alone. As has been discussed by others and us [60-62], the expression of these symptoms does not uniformly fit into neat, Western derived categorical constructs, emphasizing the need to know the person in the midst of her local social context, as well as specific diagnostic criteria.

### Limitations

Despite the importance of these findings, we recognize that our study had several limitations. There are other concerns to be mentioned. As a field survey, we were only able to approach potential participants who were home during the day. This not only omitted migrant workers living in distant cities, but also may have excluded locals who worked day-jobs away from home. Our study was cross-sectional, thus precluding mechanistic or causal analyses, even as we examined the data in ways that can provide direction for future prospective investigations. Nonetheless, the link between perceived social support and depression is consistent with the buffer theory [15]. There were two concerns we needed to address. First was that as we applied CES-D that was not a diagnostic tool with a cutoff point at 16, which was set to screen for distress and depressed symptoms. Second was that even though the CES-D has been widely adopted in China, its cut-off points have not been established in China. However, there was a large epidemiological study focusing on the validation of CES-D in China that reported that the Chinese version of CES-D showed good reliability and validity, and this study recommended that the cut-off point at 16 was applicable in China as well [63]. However, appropriate formal validations of the cut-off point of CES-D against a golden-standard diagnostic psychiatric interview have still not been completed. Hence, the high prevalence of depression in this study, compared to other studies applying diagnostic tools, could be a result of adopting the cutoff at 16 for the CES-D. However, given the nascent stage of depression research in China, this study’s findings are important despite these limitations.

We also note that our study depended on self-report measures, where perceptions of well-being or depression are subject to reporting biases [64]. Recent evidence suggests that perceived social support is more important than received social support; however, mindful of our concern [65-67], we cannot discern cause from effect. For example, we found that women who reported worse economic or health status were more likely to be depressed, with ORs of 1.63 and 1.87 respectively, a finding consistent with prior research [68]. Without a prospective longitudinal study, it is not possible to define independent and dependent variables or explore possible bi-directional relationships.

## Conclusions

This study demonstrates that women in rural China are facing significant symptoms of depression. Among these women, individuals with higher levels of social support were less likely to be depressed. Our results suggest that future studies on preventing or overcoming depression and distress will benefit from enhancing perceived and instrumental social support.

## Abbreviations

CIDI: The composite international diagnostic interview
MD-NOS: Mood disorder not otherwise specified
NHFPC: The national health and family planning commission of the people’s republic of China
DSM: The diagnostic and statistical manual of mental disorders
CES-D: The center for epidemiologic studies depression scale
DSSI: The duke social support index
OR: Odds ratio
ANOVA: Analysis of variance
GDP: Gross domestic product
